# Biofilm Production, Distribution of *ica* Genes, and Antibiotic Resistance in Clinical Coagulase-Negative Staphylococci Isolates

**DOI:** 10.3390/antibiotics14121215

**Published:** 2025-12-03

**Authors:** Neşe Erdoğan Deniz, Yüksel Akkaya, İbrahim Halil Kılıç

**Affiliations:** 1 Department of Biology, Institute of Science, Gaziantep University, 27310 Gaziantep, Turkey; nese27erdogan@gmail.com; 2 Department of Medical Microbiology, Hamidiye Faculty of Medicine, University of Health Sciences, 34668 İstanbul, Turkey; yuksel.akkaya@sbu.edu.tr

**Keywords:** coagulase-negative staphylococci, biofilm, *ica* genes, antibiotic resistance, methicillin

## Abstract

**Backgrounds/Objectives:** This study aimed to quantify biofilm production and characterize the distribution of the biofilm-associated *ica* genes (*icaA*, *icaD*, *icaB*, *icaC*, *icaR*) in coagulase-negative staphylococci (CoNS) isolates, and to assess the association between these genes and antibiotic resistance profiles. **Methods:** A total of 121 CoNS isolates collected at Ümraniye Training and Research Hospital between 1 January and 30 August 2024 were identified by VITEK 2 Compact and MALDI-TOF MS. Biofilm production was quantified using the microtiter plate assay, and the presence of *ica* genes was determined by quantitative real-time PCR (qPCR). Antimicrobial susceptibility testing (AST) was performed with the VITEK 2 Compact (bioMérieux), and minimum inhibitory concentrations (MICs) were interpreted according to EUCAST criteria. **Results:** *S. epidermidis* was found to have the highest biofilm production capacity among the CoNS isolates, followed by *S. haemolyticus*. The *icaA* gene was detected in 99.17% of isolates, followed by *icaR* (70.24%), *icaD* (55.37%), and both *icaB* and *icaC* (28.92% each). The highest resistance rates were observed for oxacillin (85.8%) and erythromycin (85.1%), while all isolates remained susceptible to linezolid, daptomycin, and vancomycin. **Conclusions:** The high prevalence of *ica* genes in CoNS isolates indicates that biofilm formation plays a critical role in the pathogenesis of these species. The findings reveal that CoNS have a strong biofilm production potential, which is a decisive factor in their pathogenicity. However, the high methicillin resistance rates emerge as one of the main factors limiting the effectiveness of current treatment options. Therefore, future studies need to focus on the development of anti-biofilm approaches and alternative therapeutic strategies.

## 1. Introduction

Staphylococci are a broad group of medically relevant Gram-positive bacteria that are typically observed microscopically in grape-like clusters and are characterized by catalase positivity [1]. These organisms are commonly found as part of the normal flora of the upper respiratory tract, skin, and other mucosal surfaces in humans and animals [2]. Based on coagulase production, staphylococci are classified into two main groups: coagulase-positive staphylococci (CoPS) and coagulase-negative staphylococci (CoNS). Within these groups, *Staphylococcus aureus* is regarded as the principal pathogen among CoPS, whereas *S. epidermidis* is considered a dominant commensal in the CoNS group and is also recognized as an opportunistic pathogen [3,4].

Coagulase-negative staphylococci (CoNS) comprise more than half of all known staphylococcal species and include approximately 50 species. *S. epidermidis* is considered the predominant species in the skin microbiota, while *S. capitis*, *S. haemolyticus*, and *S. warneri* are also frequent colonizers and have been associated with infections that may progress to sepsis in susceptible hosts. This ecological niche of CoNS is regarded as a factor that increases the risk of contamination and subsequent infection, particularly in surgical sites and during invasive medical procedures [3,5,6,7].

The clinical relevance of CoNS has largely been attributed to antimicrobial resistance and the capacity for biofilm formation. The coexistence of these traits has been linked to their role as nosocomial opportunistic pathogens, particularly in immunocompromised individuals and in patients with long-term indwelling medical devices [8]. Antimicrobial resistance is widely regarded as a global public health concern and has been projected to result in millions of deaths per year by 2050 [9]. The extensive empirical and prophylactic use of antibiotics is thought to have contributed to the selection of methicillin-resistant and multidrug-resistant CoNS populations, which may reduce the effectiveness of agents such as flucloxacillin that have traditionally been used in treatment [8,10]. Increasing resistance rates are likely to complicate antimicrobial selection and may negatively influence clinical outcomes.

Biofilm formation is considered an important virulence-associated trait in coagulase-negative staphylococci (CoNS) [4]. The genetic basis of biofilm formation is known to vary across species and even among strains of the same species. The *icaADBC* operon is regarded as a major determinant of polysaccharide-mediated biofilm formation: *icaA* and *icaD* participate in the biosynthesis of polysaccharide intercellular adhesin (PIA), *icaC* contributes to translocation of the polymer across the cytoplasmic membrane, and *icaB* is involved in partial deacetylation at the cell surface. *icaR*, which is transcribed in the opposite direction, encodes a TetR family repressor that binds to the promoter region of the *icaADBC* operon and suppresses its transcription; however, the higher-level regulatory networks acting on *icaR* have not been fully defined [11,12].

In addition to PIA-dependent mechanisms, PIA-independent biofilm formation has also been described. In these pathways, surface adhesion and subsequent biofilm accumulation in CoNS can be mediated by proteins such as accumulation-associated protein (Aap) and by extracellular DNA [5]. This molecular flexibility, combined with clinical resistance phenotypes, significantly complicates the management of CoNS infections.

Biofilms play a crucial role in the development of infections and contribute to the progression of many chronic diseases. In particular, biofilm formation in CoNS is one of the primary reasons for their pathogenicity and antimicrobial resistance [13,14]. Biofilm-associated infections are typically persistent and difficult to eradicate, underscoring the need for a better understanding of biofilm formation and maintenance mechanisms.

This study aimed to determine the distribution of *ica* genes (*icaA*, *icaD*, *icaB*, *icaC* and *icaR*) involved in biofilm formation in CoNS species and to evaluate the impact of these genes on biofilm production capacity and antimicrobial resistance profiles. By doing so, we aimed to better understand the biofilm-mediated pathogenic mechanisms of CoNS infections and to contribute to the development of effective treatment strategies.

## 2. Materials and Methods

### 2.1. Bacterial Isolates

The study included coagulase-negative staphylococci (CoNS) samples submitted to the Microbiology Laboratory of Ümraniye Training and Research Hospital from various clinics between 1 January 2024 and 30 August 2024. A total of 121 CoNS isolates were included. Species identification and antimicrobial susceptibility testing were performed by conventional culture-based methods. These isolates consisted of 42 *Staphylococcus epidermidis*, 14 *Staphylococcus haemolyticus*, 47 *Staphylococcus hominis*, and 18 *Staphylococus capitis*.

The bacterial isolates were stored at −20 °C until testing. Biofilm production was assessed in all 121 isolates using the microtiter plate assay. In the same set of isolates, the presence of the biofilm-associated *ica* genes (*icaA*, *icaD*, *icaB*, *icaC* and *icaR*) was investigated.

Clinical and laboratory data for all specimens submitted within the study period were retrieved from the hospital information management system. The study was approved by the Ümraniye Training and Research Hospital Clinical Research Ethics Committee (decision dated 12 December 2024, approval no. 408).

### 2.2. Bacterial Identification

Specimens sent from various clinics to the Microbiology Laboratory of Ümraniye Training and Research Hospital were initially inoculated onto 5% sheep blood agar (BD, Franklin Lakes, NJ, USA), chocolate agar (BD, USA), and MacConkey agar (Becton Dickinson, USA), and incubated aerobically at 35–37 °C for 24–48 h. Smears prepared from specimens showing growth were examined by Gram staining. Species-level identification was performed using automated systems—Vitek 2 Compact (bioMérieux, Marcy-l’Étoile, France) and MALDI-TOF MS/Vitek MS (bioMérieux, France)—according to the manufacturer’s protocols.

### 2.3. Antimicrobial Susceptibility Testing

Antimicrobial susceptibility profiles of the Staphylococcus isolates were determined using the Vitek 2 Compact system (bioMérieux, Marcy l’Étoile, France). Results were obtained after 8–12 h of incubation, and MIC values were interpreted according to the criteria of the European Committee on Antimicrobial Susceptibility Testing (EUCAST).

### 2.4. Microtiter Plate (MTP) Assay for Detecting Biofilm Formation Capacity

Bacterial biofilm production was investigated using the microtiter plate method adapted from the studies of Christensen et al., 1985 [15], and Ghaioumy et al., 2021 [16]. All CoNS isolates were cultured on 5% sheep blood agar (Merck KGaA, Darmstadt, Germany) at 37 °C for 24 h. Then, colonies were suspended in sterile physiological saline and adjusted to a turbidity of 0.5 McFarland. Sterile flat-bottomed 96-well polystyrene microplates were prepared by adding 180 μL of trypticase soy broth (TSB, Merck, Germany) supplemented with 1% glucose to each well, and 20 μL of the 0.5 McFarland bacterial suspension was added to each well. For each clinical isolate, three technical replicates were included on the same plate. On every microplate, Staphylococcus aureus ATCC 25923 was included as a strong biofilm-forming positive control, and TSB supplemented with 1% glucose was used as a negative control. After incubation at 37 °C for 24 h, the plates were gently inverted to remove the contents, and each well was washed three times with 200 μL of sterile phosphate-buffered saline (PBS) to remove planktonic cells. The plates were inverted and allowed to dry, then 200 μL of methanol was added to each well and incubated at room temperature for 10 min to fix the biofilm. After removing the methanol and air-drying, 200 μL of 1% crystal violet was added to each well and left for 15 min. The plates were then rinsed by gently washing three times with 200 μL of sterile physiological saline to remove excess stain. After air drying, 200 μL of 95% ethanol was added to each well and incubated for 30 min at room temperature (to prevent evaporation, the plates were covered with film during this step). The absorbance of crystal violet dissolved in ethanol, which reflects biofilm biomass, was measured at 570 nm using a spectrophotometer (Multiskan Go, Thermo Scientific, Waltham, MA, USA). As a negative control, 200 μL of TSB + 1% glucose was added to wells designated for background optical density (ODnc). As a positive control, wells received 180 μL of TSB + 1% glucose and 20 μL of a 0.5 McFarland suspension of Staphylococcus aureus ATCC 25923 (a known biofilm-producing strain). Biofilm results were evaluated quantitatively. ODc (Optical density cut-off point) was calculated with the formula ODc = ODnc + (3 × standard deviation), where ODnc is the average absorbance of three negative control wells. The OD value (absorbance) was calculated separately for each microplate. Strains were classified as follows based on their OD in the assay (Christensen et al., 1985 [15]):

- OD ≤ ODc ⇒ no biofilm production (biofilm-negative)
- ODc < OD ≤ 2 ODc ⇒ weak biofilm producer
- 2 ODc < OD ≤ 4 ODc ⇒ moderate biofilm producer
- 4 ODc < OD ⇒ strong biofilm producer

The microtiter plate biofilm assay was performed in three independent experimental runs to confirm the reproducibility of the results.

### 2.5. DNA Isolation and Real-Time PCR

Genomic DNA was extracted from CoNS strains using the PureLink™ Genomic DNA Mini Kit (Thermo Fisher, K182002, Waltham, MA, USA) according to the manufacturer’s instructions. After DNA isolation, the presence of the *icaA*, *icaD*, *icaB*, *icaC*, and *icaR* genes was investigated using a Real-Time PCR system (Thermo Fisher StepOnePlus, USA). For fluorescent staining, PowerUp™ SYBR™ Green Master Mix for qPCR (Thermo Fisher, A25742, USA) was used. Real-time PCR was performed with an initial denaturation at 95 °C for 10 min, followed by 40 cycles of 95 °C for 15 s and 60 °C for 1 min. Specific primers targeting the *icaA*, *icaD*, *icaB*, *icaC*, and *icaR* genes were used (see Table 1 for sequences). DNA from *S. epidermidis* ATCC 35984 was used as the positive control, and a no-template reaction mix was included as the negative control. Each sample was analyzed in triplicate (technical replicates), and a no-template control was included in every run to monitor for contamination.

### 2.6. Statistical Analysis

Data analysis was performed using IBM SPSS Statistics version 27 (IBM Corp., Armonk, NY, USA). The relationship between biofilm formation and *ica* genes was assessed using Pearson’s correlation test. Additionally, comparisons of the carriage rates of these genes between groups were made using the Mann–Whitney U test. To evaluate the association between bacterial species and biofilm production strength, the chi-square test of independence was used. A *p*-value < 0.05 was considered statistically significant for all analyses.

## 3. Results

### 3.1. Distribution of Isolates

**Figure 1:**
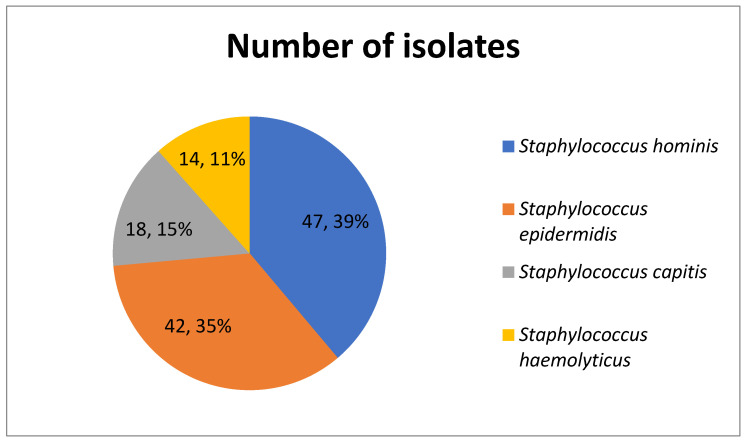
Distribution of CoNS isolates by species.

A total of 121 coagulase-negative staphylococci (CoNS) clinical isolates were analyzed in this study. These comprised 42 (35%) *S. epidermidis*, 14 (11%) *S. haemolyticus*, 47 (39%) *S. hominis*, and 18 (15%) *S. capitis*. Figure 1 shows the distribution of the CoNS isolates by species.

These 121 CoNS isolates were obtained from various clinical specimens (Table 2).

### 3.2. Antimicrobial Susceptibility

The antimicrobial susceptibility profiles of the CoNS isolates are summarized in Table 3. A total of 121 clinical isolates belonging to four different CoNS species (*S. hominis*, *S. epidermidis*, *S. capitis*, *S. haemolyticus*) were tested for susceptibility to various antibiotics.

In this study, across all CoNS species, glycopeptide and broad-spectrum antibiotics (vancomycin, linezolid, tigecycline, nitrofurantoin, daptomycin) exhibited the highest susceptibility rates. Vancomycin, tigecycline, and nitrofurantoin showed nearly 100% effectiveness against all isolates, and the susceptibility rate to linezolid was determined to be 96.7%. The preservation of high susceptibility to these agents indicates that they remain effective and reliable options for the treatment of CoNS infections. In contrast, oxacillin testing revealed widespread methicillin resistance in all CoNS species. Additionally, the resistance rate to levofloxacin was found to be above 70%, and marked differences in susceptibility were observed among species for antibiotics such as tetracycline and trimethoprim.

Overall, the vast majority of CoNS isolates exhibited multi-antibiotic resistance. Nonetheless, linezolid, vancomycin, tigecycline, and nitrofurantoin emerged as the most effective agents in treatment, maintaining high activity against these isolates.

### 3.3. Biofilm Production

**Figure 2:**
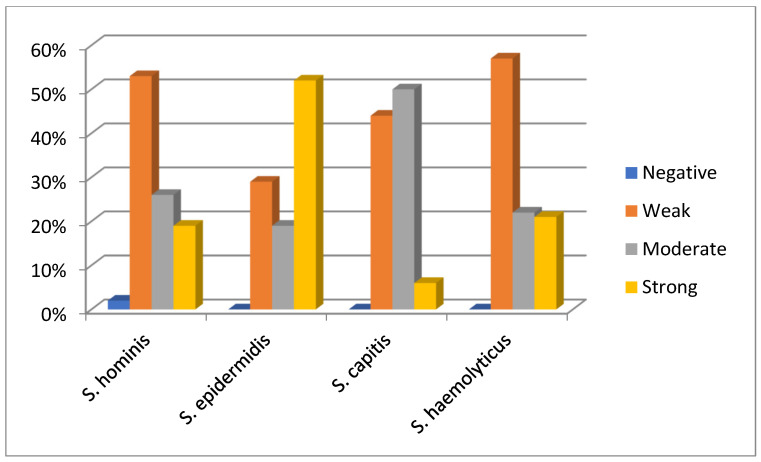
Distribution of biofilm production levels (negative, weak, moderate, strong) in clinical isolates of four different CoNS species (*Staphylococcus hominis*, *S. epidermidis*, *S. capitis*, *S. haemolyticus*). Percentage values are calculated based on the total number of isolates for each species.

The biofilm production levels of CoNS isolates differed among species. Among the 47 *S. hominis* isolates, 1 (2%) was biofilm-negative (no biofilm production), 25 (53%) were weak biofilm producers, 12 (26%) were moderate biofilm producers, and 9 (19%) were strong biofilm producers. Among the 42 *S. epidermidis* isolates, 12 (29%) were weak biofilm producers, 8 (19%) moderate, and 22 (52%) strong biofilm producers. For the 18 *S. capitis* isolates, 8 (44%) were weak, 9 (50%) moderate, and 1 (6%) was a strong biofilm producer. Among the 14 *S. haemolyticus* isolates, 8 (57%) were weak, 3 (22%) moderate, and 3 (21%) were strong biofilm producers. Figure 2 illustrates the distribution of biofilm production levels (negative, weak, moderate, strong) for the isolates of the four CoNS species.

In comparisons between species, the highest proportion of strong biofilm production was observed in *S. epidermidis* (52% of its isolates), followed by *S. haemolyticus* (~21% of its isolates). Statistical analysis showed that *S. epidermidis* had a significantly higher tendency for strong biofilm production compared to *S. capitis* and *S. hominis* (*p* < 0.05), while the difference between *S. epidermidis* and *S. haemolyticus* was not statistically significant (*p* = 0.096).

### 3.4. Biofilm-Related Genes

In this study, the distribution of genetic determinants involved in biofilm formation was examined in the 121 CoNS isolates. In this context, the presence of the *icaA*, *icaD*, *icaB*, *icaC* and the regulatory *icaR* gene was evaluated by Real-Time PCR. Analyses revealed differences in gene positivity rates between species, and showed that these genes were detected more frequently in isolates with a high biofilm production potential. Of the 121 CoNS isolates included in the study, 85 (70.24%) were positive for the *icaR* gene, 120 (99.17%) for *icaA*, 67 (55.37%) for *icaD*, 35 (28.92%) for *icaB*, and 35 (28.92%) for *icaC*. Table 4 presents the distribution of these biofilm-associated genes among the CoNS isolates.

In all species, the *icaA* gene was detected at the highest frequency, whereas the *icaB* and *icaC* genes were present at the lowest and equal frequencies. This suggests that the *icaA* gene is a key determinant in biofilm formation in CoNS.

From the center outward, the rings represent the genes and the biofilm production level for each isolate (*S. hominis*, *S. epidermidis*, *S. capitis*, *S. haemolyticus*). SE: *S. epidermidis*; SH: *S. hominis*; SC: *S. capitis*; Sha: *S. haemolyticus*. Color intensity indicates the presence/level of each gene and the biofilm phenotype. In the outermost biofilm ring, green indicates high biofilm production, light green indicates moderate biofilm, and purple indicates low biofilm. In the *ica* gene rings, purple indicates presence of the gene and red indicates absence of the gene.

**Figure 3:**
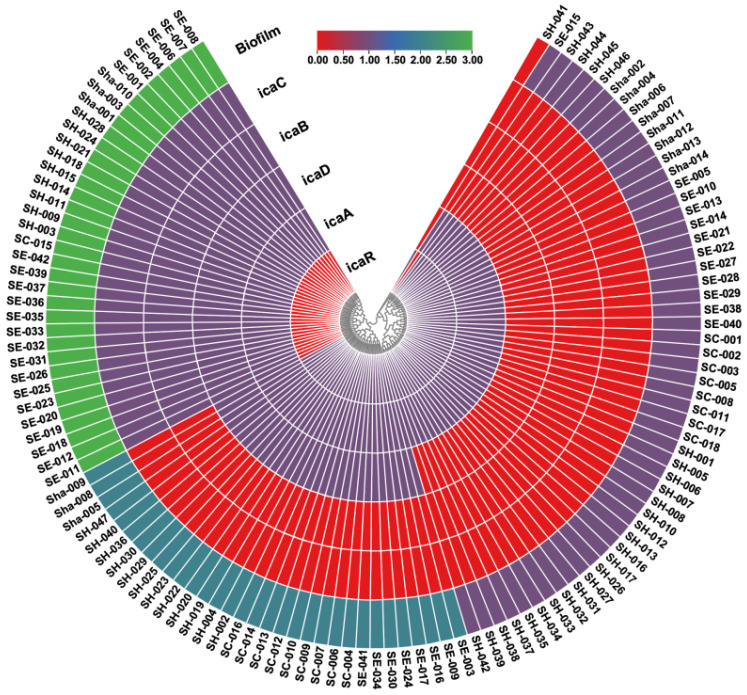
Circular heatmap showing the distribution of biofilm activity and biofilm-related genes (*icaR*, *icaA*, *icaD*, *icaB*, *icaC*) in a total of 121 CoNS isolates.

As shown in Figure 3, the *icaA* gene was highly positive in the vast majority of isolates, whereas the *icaR* and *icaD* genes showed a more variable distribution. The biofilm phenotypic strength also exhibited heterogeneity among different species (outer ring of Figure 3).

In this study, among the 121 CoNS isolates, only the *S. hominis*-41 (SH-041) isolate was found to be negative for the *icaA* gene (Figure 3). This *S. hominis* strain, obtained from a blood culture, is the only isolate in our series that did not produce biofilm, and in this strain only the *icaR* gene was detected. As shown in Figure 3, apart from the *S. hominis*-41 (SH-041) and *S. epidermidis*-015 (SE-015) isolates, the remaining isolates in our study clustered into three main groups based on the presence of the *icaR*, *icaA*, *icaD*, *icaB*, and *icaC* genes. In the absence of the *icaR* gene, it was observed that—except for the *S. epidermidis*-015 isolate—all other isolates had the *icaADBC* gene cluster active and these isolates displayed a strong biofilm phenotype. As is known, the *icaR* gene is the negative regulator of the *icaADBC* operon and plays a suppressive role in biofilm formation. In the second cluster, isolates carrying both the *icaR* and *icaAD* genes showed moderate biofilm formation, whereas in the third cluster, isolates containing *icaA* and *icaR* (but lacking other *ica* operon genes) exhibited weak biofilm production. These findings demonstrate a direct relationship between the presence of single or multiple components of the *icaADBC* gene cluster and the level of biofilm production. For instance, the *S. epidermidis*-015 (SE-015) isolate contained only the *icaA* gene and was found to produce a weak biofilm. Considering the overall distribution (refer back to Table 3), out of 121 isolates, 85 (70.24%) harbored *icaR*, 120 (99.17%) harbored *icaA*, 67 (55.37%) harbored *icaD*, 35 (28.92%) harbored *icaB*, and 35 (28.92%) harbored *icaC* genes.

Analyses indicated that gene positivity rates differed among species and that isolates with higher biofilm production potential tended to carry these genes more frequently. To statistically evaluate the relationship between biofilm production level and the presence of *ica* genes, a Pearson correlation analysis was performed (Table 5). The analysis showed that the presence of the *icaR* gene had a strong inverse correlation with biofilm production level (r = −0.875, *p* = 0.001), whereas the presence of *icaD*, *icaB*, and *icaC* genes was positively correlated with biofilm production level. No significant correlation was found for the *icaA* gene (present in nearly all isolates) with biofilm level (r = 0.091, *p* = 0.319). However, the prevalence of the *icaA* gene was significantly higher than that of the other *ica* genes (*p* < 0.001). Overall, these results suggest that the *icaA* gene plays a fundamental key role in biofilm formation, whereas the *icaR* gene functions as a negative regulator of biofilm synthesis.

For further analysis, the antibiotic susceptibility rates in strong, moderate, and weak biofilm-producing CoNS isolates were compared (Table 6). No statistically significant differences were found in the susceptibility profiles among the strong, moderate, and weak biofilm groups (*p* > 0.05). In general, when comparing the strong, moderate, and weak biofilm-producing groups, the antibiotic susceptibility patterns were similar. Independent of biofilm production level, high susceptibility was maintained to vancomycin, linezolid, daptomycin, and tigecycline, whereas susceptibility to oxacillin, levofloxacin, erythromycin, and fusidic acid was limited—isolates exhibited high resistance to these agents. Our data indicate that no meaningful relationship exists between the degree of biofilm production and susceptibility or resistance to the antibiotics tested.

## 4. Discussion

Coagulase-negative staphylococci (CoNS) are microorganisms that reside commensally on areas such as human skin and the anterior nares, but due to their opportunistic pathogenic nature, they can cause both hospital- and community-acquired infections [18,19]. Their ability to form biofilms and their resistance to multiple classes of antibiotics increase the clinical importance of these bacteria. *S. epidermidis*, by virtue of these traits, stands out as a leading cause of nosocomial infections [19].

In the literature, among CoNS strains, *S. epidermidis* and *S. haemolyticus* in particular have been reported to possess the highest pathogenic potential in healthcare-associated infections. These infections include those acquired during hospital stay, surgical interventions, or the use of medical devices such as catheters, prostheses, and shunts [4,20]. Additionally, CoNS isolates have shown a marked increase in antimicrobial resistance rates over the years, which has become a significant issue in the treatment of hospital infections [4].

In our study, oxacillin (methicillin) testing revealed that methicillin resistance was common in all CoNS species. The majority of *S. hominis*, *S. epidermidis*, *S. capitis*, and *S. haemolyticus* isolates were methicillin-resistant (MR-CoNS), showing high resistance rates of 89.1%, 81%, 88.9%, and 85.7%, respectively. Consistent with the literature, *S. epidermidis* and *S. haemolyticus* have frequently been associated with high methicillin resistance rates [21,22,23,24]. In our study, a significant portion of *S. epidermidis* and *S. haemolyticus* isolates were also found to be resistant to levofloxacin, erythromycin, clindamycin, and tetracycline, paralleling findings from previous studies [4,25]. Consequently, all isolates in our study exhibited high overall rates of antibiotic resistance; the highest resistance was observed for oxacillin (84.3%), erythromycin (85.1%), and fusidic acid (83.4%). In contrast, the resistance rate to clindamycin (approximately 69.4%) was comparatively lower.

Linezolid, an oxazolidinone antibiotic, remains an important option for treating multi-resistant staphylococcal infections. Although there have been reports of linezolid resistance emerging in some CoNS species [26,27], overall susceptibility to linezolid has generally remained high. In our study as well, the susceptibility rate to linezolid was found to be very high, with 95.8% of isolates being susceptible. This finding indicates that linezolid is still an effective and reliable alternative for the treatment of CoNS infections in our setting.

Overall, these findings indicate that multidrug resistance is frequent among biofilm-forming CoNS isolates, but that the degree of biofilm production (strong, moderate, or weak) was not associated with major differences in susceptibility patterns in our cohort. High resistance to agents such as oxacillin, levofloxacin, erythromycin, and fusidic acid was observed across all biofilm categories, whereas susceptibility to vancomycin, linezolid, daptomycin, and tigecycline remained largely preserved. Together with the limited number of biofilm-negative isolates, this suggests that biofilm formation and resistance frequently coexist; however, a simple linear relationship between biofilm intensity and resistance level could not be demonstrated.

A biofilm is defined as a microbial community attached to biotic or abiotic surfaces, surrounded by an extracellular polymeric matrix, and exhibiting increased resistance to antimicrobials [28]. Coagulase-negative staphylococci, through their capacity to produce biofilms that confer resistance to antibiotics and host immune responses, rank among the foremost pathogens in device-associated infections [29]. It is reported that approximately 80% of hospital-acquired infections are associated with biofilm-forming bacteria. Biofilm-forming microorganisms can be up to 1000 times more resistant to antibiotics than their planktonic counterparts [30,31,32]. Moreover, mutations play a critical role in the emergence of antibiotic resistance in staphylococci; for instance, the study by Ryder et al., 2012 [33] demonstrated that *S. epidermidis* biofilm cells undergo mutations at about four times the rate of planktonic cells, which was particularly related to increased rifampicin resistance [33,34].

According to our findings, 0.8% of the 121 CoNS isolates were biofilm-negative, 43.8% were weak biofilm producers, 26.4% were moderate, and 28.9% were strong biofilm producers. When compared across species, the highest rate of strong biofilm production was observed in *S. epidermidis* at 52%, followed by *S. haemolyticus* at 21%. This observation is consistent with the results reported by Dengler Haunreiter et al., 2019 [35], which indicated that *S. epidermidis* develops evolutionary adaptations during interaction with the host that increase antibiotic tolerance and strengthen its biofilm-forming capacity. In our results, the fact that *S. epidermidis* had the highest proportion of strong biofilm producers underscores the clinical importance of this species and supports the findings in the literature.

Biofilm formation fundamentally relies on the activity of the *icaADBC* operon in CoNS, which is responsible for the synthesis of the extracellular matrix, particularly the production of polysaccharide intercellular adhesin (PIA) [36]. In this system, the *icaA* gene encodes an N-acetylglucosamine transferase enzyme, and the *icaD* gene acts as a chaperone ensuring proper folding of the IcaA protein; the product of the *icaC* gene is involved in transporting PIA to the cell surface, while *icaB* functions in the deacetylation of PIA, facilitating its attachment to the cell surface [37]. Therefore, detection of the genes that contribute to biofilm formation is seen as a determining factor in pathogenesis, and investigating these genes is highly important [38].

In our study, we evaluated the presence of the *ica* operon genes involved in biofilm formation in clinical CoNS isolates, and we found a high prevalence of these genes (especially *icaA* and *icaR*), as detailed in the results. Our findings can be compared to those of previous studies: Altunova and Kılıç, 2025 [30] detected the *icaA* gene in 87.7% and the *icaD* gene in 92.3% of clinical CoNS isolates. Phillip et al., 2023 [39] reported that among 65 CoNS isolates associated with urinary tract infections, the most dominant virulence genes were *icaC* (46.5%) and *icaA* (13.9%). Azmi et al., 2019 [40] found that in *S. aureus* isolates, the biofilm production distribution was 21% strong, 46.4% moderate, 32.6% weak, and all isolates were positive for *icaA* and *icaD*. In Iran, Alibegli et al., 2025 [38] observed *icaA*, *icaB*, *icaC*, and *icaD* gene positivity rates of 90%, 92%, 92%, and 94%, respectively, in *S. aureus* isolates. In our research on 121 CoNS isolates, we detected the *icaA* (99.17%) and *icaR* (~70–71%) genes at the highest frequencies, *icaD* (55.37%) at a moderate level, and *icaB* and *icaC* at lower frequencies (28.92%) (see Table 4 and Section 3). These rates are broadly in line with, or somewhat higher than, those reported in other studies for similar genes, underscoring the strong biofilm-forming genetic potential of our CoNS isolates.

Notably, in our findings the *S. epidermidis*-015 isolate lacked the *icaR* gene and contained only *icaA*, corresponding with a weak biofilm phenotype. This situation suggests that the strain might have been introduced from a different source into the population or, similar to the observations of Hoang et al. (2019) [41], an alternative mechanism (such as the *tcaR* gene) might be influencing biofilm formation. Hoang et al. [41] reported that both IcaR and TcaR regulate the *icaADBC* operon in *S. epidermidis*, indicating that other regulatory pathways can impact biofilm gene expression. To elucidate these differences, more detailed studies employing advanced biotechnological methods are needed.

The literature indicates significant differences in the prevalence of *icaA*, *icaB*, *icaC*, and *icaD* genes between biofilm-positive and biofilm-negative isolates; the vast majority of biofilm-positive staphylococcal isolates carry these genes at higher rates [37,42]. Similarly, Piechota et al., 2018 [43] reported that the presence of *ica* genes was significantly associated with biofilm production, and, in particular, isolates harboring *icaADBC* or *icaABD* gene combinations exhibited higher biofilm capacity than strains containing only *icaAD*. Additionally, one study found a statistically significant difference (*p* = 0.000) between biofilm-positive and biofilm-negative isolates in terms of *icaA*, *icaB*, *icaC*, and *icaD* gene presence; over 95% of biofilm-producing isolates were reported to carry all of these genes together [38]. In that study, it was determined that weak biofilm-producing strains carried the *icaA* gene, moderate biofilm producers carried *icaA* and *icaD*, and strong biofilm producers contained *icaA*, *icaB*, *icaC*, and *icaD* genes. These findings collectively suggest that as the number of *ica* gene types increases, biofilm strength also increases, and different combinations of *ica* genes may be determinative in biofilm formation. Moreover, strains lacking the *icaR* gene still carried the *icaADBC* genes and produced strong biofilms, underscoring the role of *icaR* in the negative regulation of biofilm development. In line with this, Schwartbeck et al. [17] observed that in *S. aureus* isolates from cystic fibrosis patients, various mutations in *icaR* (the repressor of the *icaADBC* locus) were associated with mucoid variants and significantly increased PIA production. *icaR* negatively regulates icaADBC transcription by binding upstream of the operon [36]. In our study, the high detection rate of the *icaR* gene is considered an important finding indicating the presence of a critical regulatory factor in the biofilm formation mechanisms of CoNS.

In this study, the relationship between biofilm production level, *ica* genes, and antibiotic resistance profiles was evaluated. Among the 121 CoNS isolates examined, the only biofilm-negative isolate (*S. hominis*) had only the *icaR* gene positive (and lacked *icaA*, *D*, *B*, and *C*). Hospital-derived staphylococcal isolates are known to produce stronger biofilms than community-derived isolates [44]. The fact that this *S. hominis* isolate, which lacked other *ica* genes and did not form a biofilm, could be of community origin is a plausible explanation. Our correlation analysis further clarified the relationship between ica gene profiles and biofilm formation. The presence of *icaD*, *icaB* and *icaC* was positively associated with higher biofilm production levels, indicating that these genes contribute to a stronger biofilm phenotype in clinical CoNS isolates. In contrast, *icaR* showed an inverse relationship with biofilm formation, which is consistent with its role as a negative regulator of the *icaADBC* operon. Because *icaA* was detected in almost all isolates, it did not display a discriminative correlation with biofilm categories; however, its near-universal presence underscores the widespread genetic potential for PIA-mediated biofilm formation in this population (Table 4).

Grazul et al., 2023 [45] found that among *S. epidermidis*, *S. hominis*, and *S. haemolyticus* isolates, the most commonly detected gene was *icaA*. Our observations are in agreement, as we also noted that the *icaA* gene was present at a very high frequency in essentially all isolates we examined.

Kord et al., 2018 [46] demonstrated a significant relationship between the presence of *icaADBC* genes and biofilm production in *S. epidermidis* clinical isolates. However, they also reported that some *ica*-positive isolates did not produce biofilm, indicating that biofilm formation is not solely dependent on the presence of these genes; differences in gene expression and environmental factors can also shape the biofilm phenotype. In our study, despite the *icaA* gene being positive in 99.17% of isolates, the observation of weak, moderate, and strong biofilm phenotypes among these isolates supports this view, suggesting that gene presence alone does not entirely predict biofilm formation.

A limitation of our study is that we did not include an additional phenotypic method such as Congo red agar for visual confirmation of biofilm or slime production; biofilm formation was assessed solely using the quantitative microtiter plate assay.

In conclusion, our findings demonstrate that clinical CoNS isolates obtained in our setting harbor a high prevalence of biofilm-associated *icaADBC*/*icaR* genes and that a substantial proportion, particularly *S. epidermidis*, exhibit strong biofilm-forming capacity together with multidrug resistance. These observations are in line with previous work showing that the *icaADBC* operon and its regulators are key determinants of PIA-mediated biofilm formation and contribute to the pathogenic potential of staphylococci [11,36,38,41], and that biofilm-producing CoNS can persist in the hospital environment and develop resistance to multiple antimicrobial agents [8,25,47,48]. From an infection control perspective, the coexistence of biofilm production and methicillin resistance in CoNS is particularly concerning in high-risk settings such as intensive care units and wards with frequent invasive procedures, where careful device management and adherence to infection-prevention bundles are essential to reduce the risk of CoNS-associated infections [5,8,25]. Although susceptibility to vancomycin, linezolid, daptomycin and tigecycline remained high in our isolates, biofilm-associated tolerance may still compromise clinical responses to these agents [13,14], underscoring the need to integrate biofilm considerations into therapeutic decision-making. Overall, our data support the view that future management of CoNS infections should combine optimized systemic antimicrobial regimens with adjunctive anti-biofilm strategies, including the use of agents that interfere with PIA-containing matrix or modulate ica-dependent biofilm pathways, as suggested by recent experimental studies [30,41].

## Figures and Tables

**Table 1:** Primers used for PCR amplification of the *icaA*, *icaD*, *icaB*, *icaC*, and *icaR* genes.

Target Gene	Primer Sequence 5′→ 3′	Amplicon Size (bp)	Reference
*icaA*	F5′TCTCTTGCAGGAGCAATCAA R5′TCAGGCACTAACATCCAGCA	(188 bp)	Grassia, G. et al., 2024 [1]
*icaB*	F5′ATGGCTTAAAGCACACGACGC R5′TATCGGCATCTGGTGTGACAG	(526 bp)	Grassia, G. et al., 2024 [1]
*icaC*	F5′ATCATCGTGACACACTTACTAACG R5′CTCTCTTAACATCATTCCGACGCC	(934 bp)	Grassia, G. et al., 2024 [1]
*icaD*	F5′ATGGTCAAGCCCAGACAGAG R5′CGTGTTTTCAACATTTAATGCAA	(198 bp)	Grassia, G. et al., 2024 [1]
*icaR*	F5′CAATATCGATTTGTATTGTCAACTTT R5′GGTTGTAAGCCATATGGTAATTGA	(798 bp)	Schwartbeck et al., 2024 [17]

**Table 2:** Distribution of CoNS isolates according to clinical specimen type.

CoNS Species	Blood Culture *n* (%)	Catheter Blood Culture *n* (%)	Urethral Discharge Culture *n* (%)	Wound Culture *n* (%)	Other *n* (%)	Total Isolates (*n*)
*Staphylococcus hominis*	44 (93.61%)	2 (4.25%)	0 (0%)	0 (0%)	1 (2.12%)	47
*Staphylococcus epidermidis*	34 (80.95%)	5 (11.90%)	0 (0%)	3 (7.14%)	0 (0%)	42
*Staphylococcus capitis*	14 (77.78%)	4 (22.22%)	0 (0%)	0 (0%)	0 (0%)	18
*Staphylococcus haemolyticus*	10 (71.43%)	3 (21.43%)	1 (7.14%)	0 (0%)	0 (0%)	14
Total isolates	102 (84.30%)	14 (11.57%)	1 (0.83%)	3 (2.48%)	1 (0.83%)	121

**Table 3:** Percentage susceptibility profiles of clinical isolates of four different CoNS species to various antibiotics (%) (S: Susceptible, R: Resistant, I: Intermediate).

Antibiotics	*S. hominis* (*n* = 47)			*S. epidermidis* (*n* = 42)			*S. capitis* (*n* = 18)			*S. haemolyticus* (*n* = 14)			Total Isolates (*n* = 121)		
	S (%)	R (%)	I (%)	S (%)	R (%)	I (%)	S (%)	R (%)	I (%)	S (%)	R (%)	I (%)	S (%)	R (%)	I (%)
Oxacillin	10.8	89.1	0	19	81	0	11.1	88.9	0	14.3	85.7	0	14.1	85.8	0
Levofloxacin	0	70.2	29.8	0	71.4	28.6	0	88.9	11.1	0	85.7	14.3	0	75.2	24.8
Erythromycin	12.8	87.2	0	23.8	76.2	0	11.1	88.9	0	0	100	0.0	14.9	85.1	0
Clindamycin	31.1	68.8	0	38.1	61.9	0	11.1	88.9	0	21.4	78.6	0.0	29.4	70.5	0
Linezolid	97.9	2.1	0	95.2	4.8	0	94.4	5.6	0	100	0.0	0	96.7	3.3	0
Daptomycin	95.7	4.3	0	92.9	7.1	0	77.8	22.2	0	100	0.0	0	92.6	7.4	0
Vancomycin	100	0	0	100	0	0	100	0	0	100	0	0	100	0	0
Tetracycline	23.4	76.6	0	40.5	59.5	0	72.2	27.8	0	35.7	64.3	0	38.0	62.0	0
Tigecycline	100	0	0	100	0	0	100	0	0	92.9	7.1	0	99.2	0.8	0
Nitrofurantoin	100	0	0	100	0	0	100	0	0	100	0	0	100	0	0
Fusidic acid	12.8	87.2	0	23.8	76.2	0	88.9	11.1	0	7.1	92.9	0	27.3	72.7	0
Trimethoprim (TMP)	89.4	10.6	0	85.7	14.3	0	94.4	5.6	0	71.4	28.6	0	86.8	13.2	0

**Table 4:** Distribution of biofilm-associated genes (*icaA*, *icaD*, *icaB*, *icaC*, *icaR*) in CoNS isolates.

CoNS Species	No. of Isolates	*icaR* Positive *n* (%)	*icaA* Positive *n* (%)	*icaD* Positive *n* (%)	*icaB* Positive *n* (%)	*icaC* Positive *n* (%)
*S. hominis*	47	38 (80.85%)	46 (97.87%)	21 (44.68%)	9 (19.14%)	9 (19.14%)
*S. epidermidis*	42	19 (45.23%)	42 (100%)	30 (71.43%)	22 (52.38%)	22 (52.38%)
*S. capitis*	18	17 (94.44%)	18 (100%)	10 (55.56%)	1 (5.56%)	1 (5.56%)
*S. haemolyticus*	14	11 (78.57%)	14 (100%)	6 (42.86%)	3 (21.43%)	3 (21.43%)
Total	121	85 (70.24%)	120 (99.17%)	67 (55.37%)	35 (28.92%)	35 (28.92%)

**Table 5:** Correlations between biofilm production level and the presence of *ica* genes in CoNS isolates.

	*icaR*	*icaA*	*İcaD*	*icaB*	*icaC*
r	−0.875	0.091	0.898	0.875	0.875
*p*	0.001	0.319	0.001	0.001	0.001

**Table 6:** Antibiotic susceptibility rates in CoNS isolates producing strong, moderate, and weak biofilms (S, susceptible; R, resistant; I, intermediate).

Antibiotics	Strong Biofilm (*n* = 35)			Moderate Biofilm (*n* = 32)			Weak Biofilm (*n* = 53)		
	S	R	I	S	R	I	S	R	I
Oxacillin	8 (22.85%)	27 (77.14%)	0 (0%)	3 (9.37%)	29 (90.62%)	0 (0%)	8 (15.09%)	45 (84.90%)	0 (0%)
Levofloxacin	0 (0%)	22 (62.85%)	13 (37.14%)	0 (0%)	28 (87.50%)	4 (%12.50%)	0 (0%)	41 (77.35%)	12 (22.64%)
Erythromycin	7 (20%)	28 (80%)	0 (0%)	2 (6.25%)	30 (93.75%)	0 (0%)	8 (15.09%)	45 (84.90%)	0 (0%)
Clindamycin	17 (48.57%)	18 (51.42%)	0 (0%)	8 (25%)	24 (75%)	0 (0%)	11 (20.75%)	42 (79.24%)	0 (0%)
Linezolid	34 (97.14%)	1 (2.85%)	0 (0%)	32 (100%)	0 (0%)	0 (0%)	50 (94.33%)	3 (5.66%)	0 (0%)
Daptomycin	33 (94.28%)	2 (5.71%)	0 (0%)	28 (87.50%)	4 (12.50%)	0 (0%)	50 (94.33%)	3 (5.66%)	0 (0%)
Vancomycin	35 (100%)	0 (0%)	0 (0%)	32 (100%)	0 (%0)	0 (0%)	53 (100%)	0 (0%)	0 (0%)
Tetracycline	11 (31.42%)	24 (68.57%)	0 (0%)	13 (40.62%)	19 (59.37%)	0 (0%)	22 (41.50%)	31 (58.49%)	0 (0%)
Tigecycline	35 (100%)	0 (0%)	0 (0%)	32 (100%)	0 (0%)	0 (0%)	52 (98.11%)	1 (1.88%)	0 (0%)
Nitrofurantoin	35 (100%)	0 (0%)	0 (0%)	32 (100%)	0 (0%)	0 (0%)	53 (100%)	0 (0%)	0 (0%)
Fusidic acid	6 (17.14%)	29 (82.85%)	0 (0%)	5 (15.62%)	27 (84.37%)	0 (0%)	8 (15.09%)	45 (84.90%)	0 (0%)
Trimethoprim (TMP)	30 (85.71%)	5 (14.28%)	0 (0%)	29 (90.62%)	3 (9.37%)	0 (0%)	45 (84.90%)	8 (15.09%)	0 (0%)

## Data Availability

All data supporting the findings of this study are available within the manuscript. Additional details can be provided upon reasonable request to the corresponding author.
